# Automated rock mass condition assessment during TBM tunnel excavation using deep learning

**DOI:** 10.1038/s41598-022-05727-5

**Published:** 2022-02-02

**Authors:** Liang Chen, Zhitao Liu, Hongye Su, Fulong Lin, Weijie Mao

**Affiliations:** 1 State Key Laboratory of Industrial Control Technology, Institute of Cyber-Systems and Control, Zhejiang University, Hangzhou, 310027 China; 2 General Institute of Design and Research, China Railway Engineering Equipment Group Co., LTD, Zhengzhou, 450016 China

**Keywords:** Mechanical engineering, Computational science

## Abstract

Rock mass condition assessment during tunnel excavation is a critical step for the intelligent control of tunnel boring machine (TBM). To address this and achieve automatic detection, a visual assessment system is installed to the TBM and a lager in-situ rock mass image dataset is collected from the water conveyance channel project. The rock mass condition assessment task is transformed into a fine-grain classification task. To fulfill the task, a self-convolution based attention fusion network (SAFN) is designed in this paper. The core of our method is the discovery and fusion of the object attention map within a deep neural network. The network consists of two novel modules, the self-convolution based attention extractor (SAE) module and the self-convolution based attention pooling algorithm (SAP) module. The former is designed to detect the intact rock regions generating the attention map, and the latter is designed to improve the performance of classifier by fusing the attention map that focuses on the intact rock regions. The results of SAFN are evaluated from aspects of interpretability, ablation, accuracy and cross-validation, and it outperforms state-of-the-art models in the rock mass assessment dataset. Furthermore, the dynamic filed test show that our assessment system based on the SAFN model is accurate and efficient for automated classification of rock mass.

## Introduction

**Figure 1:**
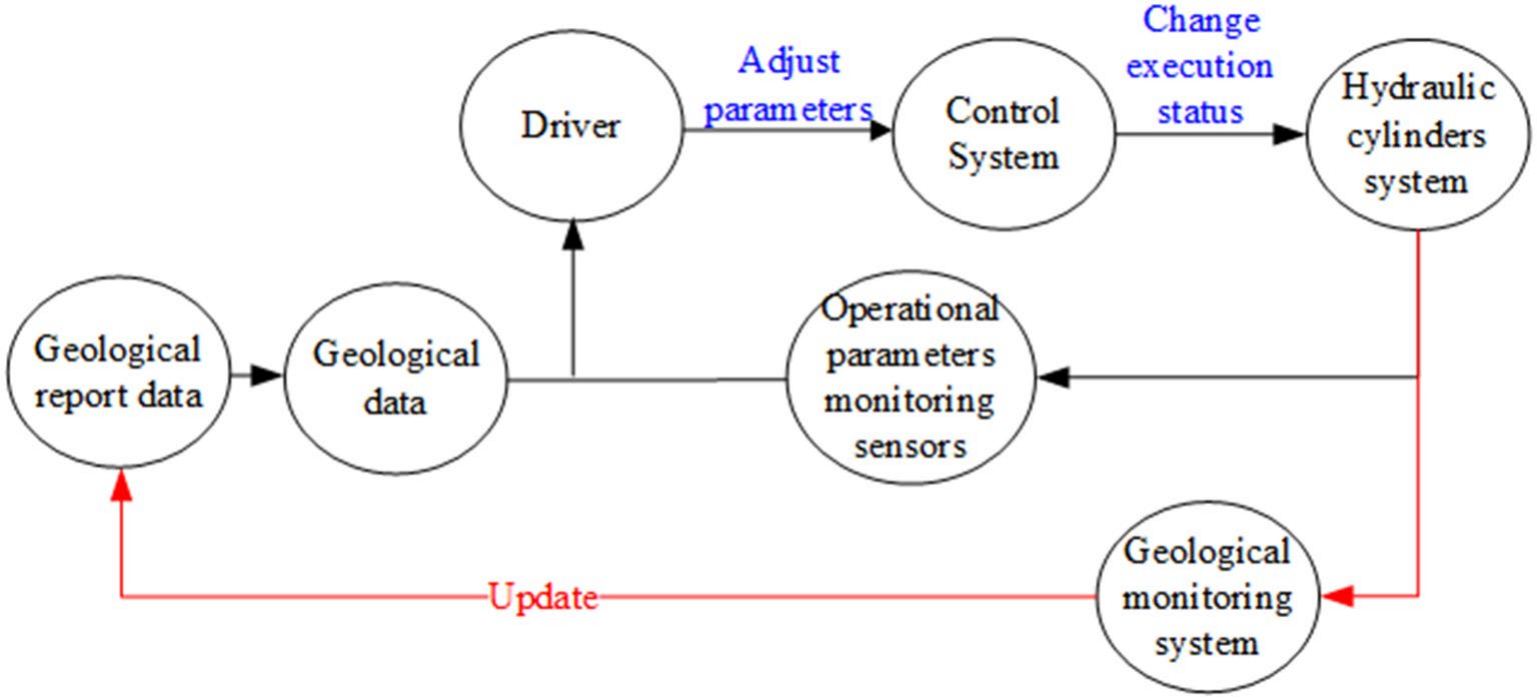
An overview of TBM driving.

The tunnel boring machine (TBM) is widely used in tunnel construction owing to its advantages in security, higher efficiency and environmental friendliness over conventional drill and blast methods^1^. Nevertheless, TBM performance is greatly affected by a wide range of geological conditions. Figure 1 illustrates that the TBM driving process. The TBM driver usually optimizes operational performance with the help of real-time operational parameters between the actuator (i.e., the cutting head) and control systems and the geological condition from the geological report. However, the geological condition in the geological investigation report is sparse density and always different from the actual rock mass types^2^. Suppose the wrong judge of the geological condition during TBM excavation, it will undoubtedly introduce serious severe challenges to the tunnel construction, such as low TBM utilization, high additional cost and even safety problems^3,4^. Therefore, to make sure about the efficient and safe tunneling process of TBM, it is very important to ensure the rock mass online assessment^5^.

Over the last decades, there have been many works on the rock mass recognition. These methods for rock mass recognition can be typically categorized into two classes: indirect prediction and direct detection.

The correlation of the TBM performance, operational parameters, and rock mass conditions substantially reflect the TBM-rock interaction mechanism. Many researchers utilize this correlation to assess rock mass conditions by numerical simulation with TBM field data. Hassanpour et al. in 2009, 2011^6,7^ propose a clustering algorithm with the field penetration index (FPI) and torque penetration index (TPI) from the data collected during the normal process to classify the condition of the rock mass. Adoko and Yagiz^8^ have established a FPI model via fuzzy inference systems which could be used to quantify the relationship between the rock mass properties and the TBM cutter load. Liu et al.^9^ developed a hybrid algorithm that combines artificial neural network (ANN) and simulated annealing (SA) for the prediction of rock mass parameters. Although these studies have made achievements for obtaining surrounding rock information from field data, there are some challenges for those methods. First, it is difficult to extract effective data from large-scale field data for those data-driven algorithms. Second, the low accuracy, stability and time consuming also limit the development of this technology.

For the direct detection, Song et al.^10^ and Su et al.^11^ propose the mechanics deduction to identify the rock mass mechanism. The stress cells should be buried in the model ground to monitor the rock stress, which is not suitable for real-time data collection during tunnel excavation. Computer vision method^12^ is one of the solutions for this problem. The convolution neural network model (CNN) is an efficient method to monitor the infrastructure health in civil engineering^13^ in an end-to-end multilayer fashion. Chen et al.^14^ obtain the rock tunnel face image to classify the rock structure of tunnel face. However, this method can only be used in manual drilling and blasting methods because the narrow space between the TBM cutterhead and tunnel face. In addition, deep learning is dependent on huge training samples^15^ and there is scarce research on the tunnel rock image at present.

In this paper, we classify the rock mass through the direct detection method. We monitor the rock excavated by the tunnel boring machine and collect a rock mass assessment dataset. This dataset is the first attempt to classify the excavated rock during TBM tunnel excavation. To fulfill the rock assessment task, we propose an assumption that if the model recognizes significant image areas and amplifies their effects while suppressing irrelevant and potentially confusing information in other regions, the classification task will benefit. Based on this assumption, the self-convolution based attention fusion network (SAFN) is proposed. The core of our method is the discovery and fusion of the object attention map within a deep neural network. We focus on the attention map not only for the region of interest (ROI) extraction, but also for improving the performance of CNN by fusing it that focuses on the in-tact rock regions in an image. The monitoring results of the system intuitively show the different types of rocks under the surface, which can provide a scientific reference for TBM control research^16^ As the first attempt to use CNNs for classification of rock mass structures captured from the excavated rock during tunnel excavation, this research made the following two contributions. First, it paves the way for other researchers to apply a higher accuracy and efficiency framework in order to classify the rock structure during tunnel excavation. Secondly, it confirms that the proposed image technique significantly improves the efficiency of conventional overall recognition.

This paper is organized as follows. The rock assessment dataset is described briefly in “Rock assessment dataset”. “Rock assessment model” describes the SFAN for rock mass assessment. “Experiment results” provides the experiment. Finally, the conclusion of this paper is drawn in “Conclusion and future work”.

## Rock assessment dataset

**Figure 2:**
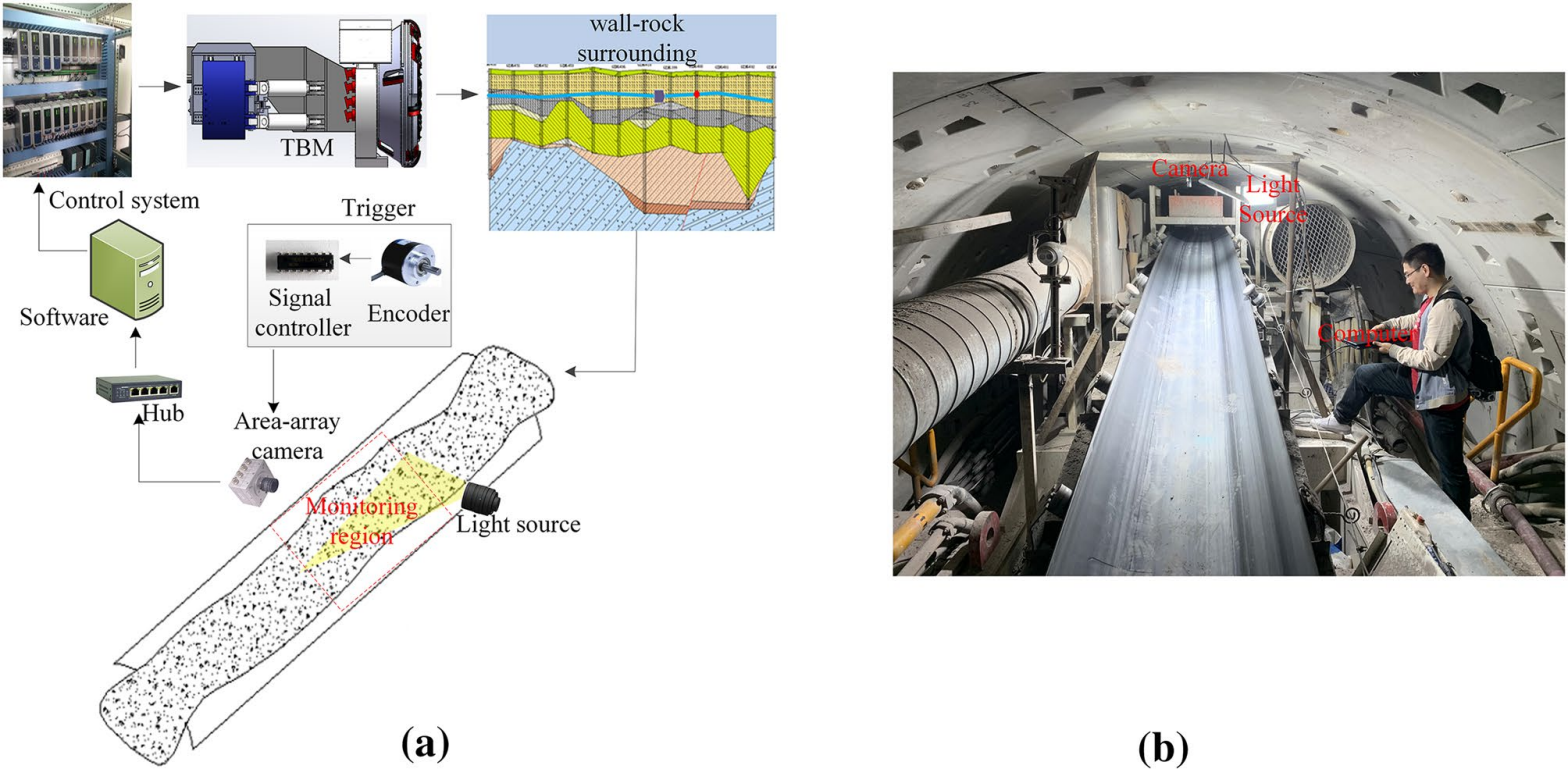
The visual assessment system. (**a**) Schematic diagram of the system. (**b**) Field test.

In general, rock mass assessment is operated by the trained TBM drivers. However, the manual operation comes at a high cost in labor and time, but the assessment results are not recorded continuously in real-time. Therefore, it is necessary to construct an automated visual assessment system to provide a reliable assessment. The schematic diagram of the assessment system is shown in Fig. 2a, which includes an area-array camera, light sources, a trigger unit, a conveyor group, and a data processing server.

The camera is HIKVISION MV-CA013-21UM with a focal length of 8 mm. The work distanced between the array camera and excavated rock is 1000 mm by shooting the cameras in the vertical orientation. The horizontal and vertical viewing angles are $55.6^{ \circ }$ and $47.6^{ \circ }$, respectively. The monitoring region covers a region of $1000\,{\text{mm}} \times 800\,{\text{mm}}$ in the conveyor belt. When the conveyor belt runs at a speed of 5 m/s, the system can capture images at a speed of 30 frames per second^17^. If the captured image has no "ghosting" details, the exposure time should be set to 200 ms. Then, the captured image is sent to the server via Ethernet and classified by the deep learning network. According to the results of the rock mass assessment, the control system of TBM can get the accurate rock mass parameters for geological adaptive control. As shown in Fig. 2b, the visual monitoring system is installed in the conveyor belt behind the segment erector, approximately 20 m away from the nose of TBM.

**Figure 3:**
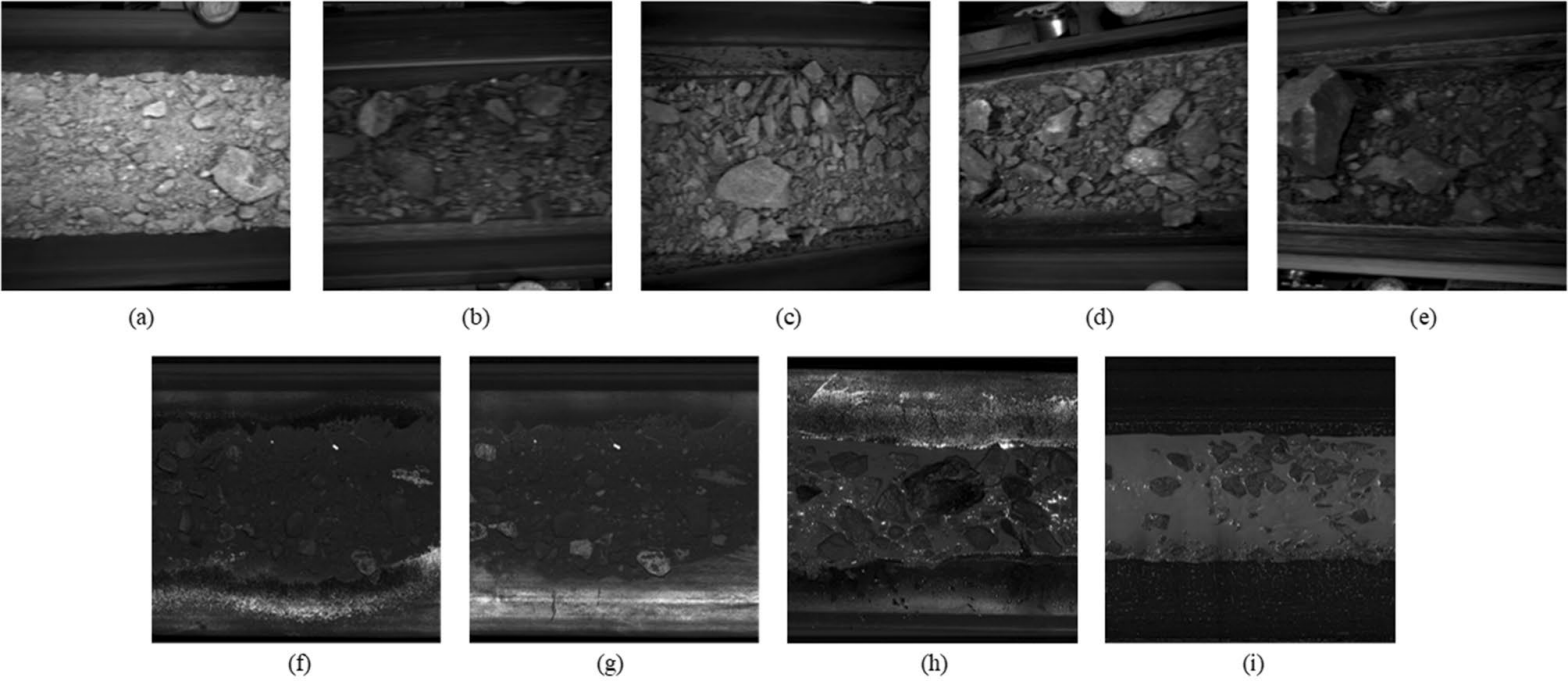
Datasets. (**a**) Granite porphyry (GP). (**b**) Conglomeratic sandstone (CS). (**c**) Argillaceous siltstone (AS). (**d**) Medium fine sandstone (MFS). (**e**) Silty mudstone (SM). (**f**) Tectonic breccia (TB). (**g**) Cryptoexplosive breccia (CB). (**h**) Argillaceous silty limestone (ASL). (**i**) Bioclastic limestone (BL).

This research team spent three months collecting the excavated rock images built in different rock mass tunnel excavation field in Hangzhou Second Source Water Conveyance Channel Project (Shanling section, Jiangnan Route), Hangzhou, China. This project, with a total length of 13.21 km, had been designed to convey and divert water from Xianlin Reservoir to Hangzhou. The size of rock images was set at $1024 \times 1024$. In this study, 12,600 nine types of rock images were collected: granite porphyry (GP), conglomeratic sandstone (CS), medium fine sandstone (MFS), argillaceous siltstone (AS), silty mudstone (SM), tectonic breccia (TB), cryptoexplosive breccia (CB), argillaceous silty limestone (ASL) and bioclastic limestone (BL) as shown in Fig. 3.

## Rock assessment model

This section focuses on designing assessment network for the rock mass. There are three challenges for our rock feature discovery.

1. Prior knowledge of the rock mass. The relatively intact rock regions retain prior knowledge of the rock veins and structure compared to the clast. It should be incorporated to learn in the classification task. The mainstream classification network can be divided into mask-guide^18,19^ methods, attention-based^20,21^ methods and global-feature-based^22,23^ methods. The mask-guided methods and the attention-based method can utilize the object information. However, the mask-guided method should label the image in large-scale collections, which is unrealistic in industry applications. We choose the attention-based method to complete the classification task, which involves an attention mechanism to extract additional prior knowledge features.

2. Attention mechanism fusion to CNN. The attention map has long been used for visual explanation^24^. The high response value is the attention location in image recognition. Few studies are attempt to improve the performance of CNN by fusing attention mechanism. We propose the SAP module integrated the attention map into the classification network.

3. Real-time requirement. The proposed visual system is used to assist TBM driving. The classification result of the rock mass is the input of the geological adaptive control. The real-time performance is critical for our system. In the existing methods, the branch structure network is widely used to extract the attention map. Our method improves the real-time performance by self-convolution operation instead of the branch deep neural network.

### Overall flow

**Figure 4:**
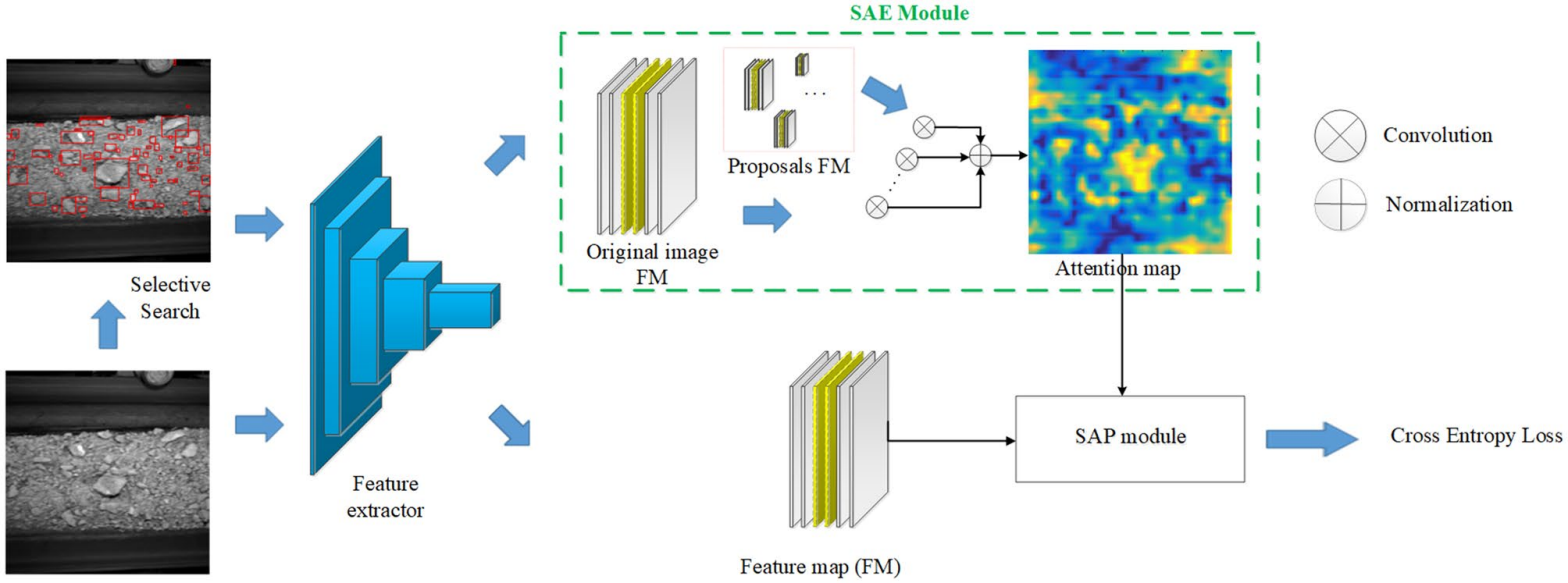
Illustration of the overall flowchart.

We propose the self-convolution based attention fusion network (SAFN) for rock mass assessment. The attention-based network architecture is highly similar to the human body consisting of three parts illustrated in Fig. 4: the baseline, extracting hidden information from the image; the limb, extracting the attention map of ROI by the self-convolution operation; and the head, fusing the attention feature to the backbone by SAP and making correct judgments on the basis of rigorous analysis.

The overall flowchart of the SAFN is depicted in Fig. 4. Our task is to predict an attention map *A* and a category *C* for each image. Given the image *I*, its proposals $R_{i}$ by selective search and the image label *B*, the feature map of the *I* is firstly extracted by the CNN baseline. Then, our model obtains the attention map *A* about the intact rock region by the self-convolution attention extractor. At last, the SAP distance is obtained by the self-convolution based attention pooling algorithm and applied to the classification network.

### Feature extractor

**Figure 5:**
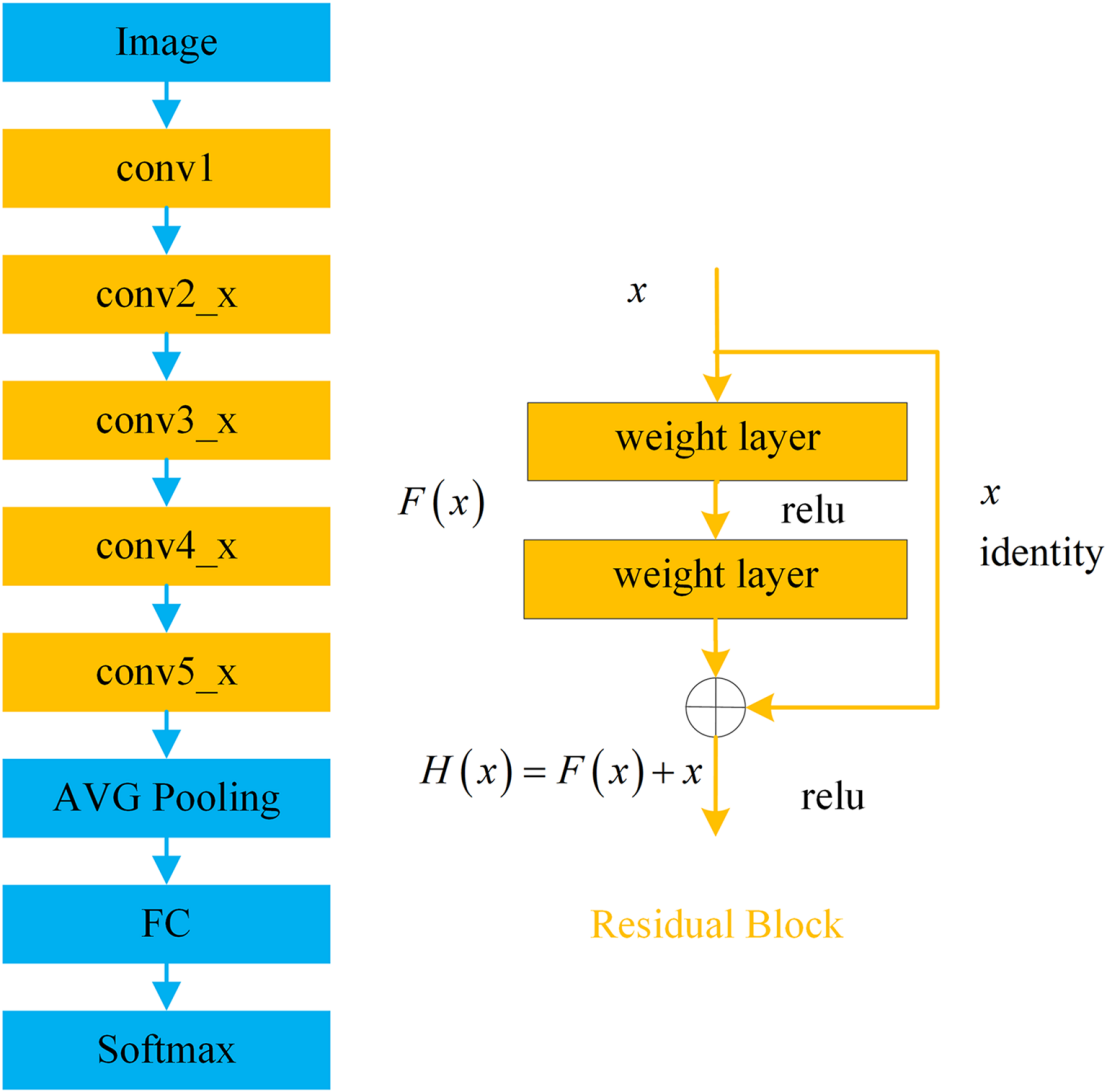
The framework of the ResNet-50.

In the attention-based network, the baseline network is the basic^25^. The deep residual learning framework (ResNet)^26^ is proposed for the degradation problem of the increased number of convolutional layers, which is one of the most popular models at present. The ResNet-50 framework is depicted in Fig. 5, which can be divided into 5 stages. The ResNet framework addressed this problem through shortcut connections, skipping one or more layers, and simply performing identity mapping. The residual block fits the residual function (1).

1 $$H\left( x \right) = F\left( x \right) + x,$$

where $x$ is the identity mapping; $H\left( x \right)$ is arbitrary desired mapping. Since the output of multiple nonlinear layers $F\left( x \right)$ degrades to zero, the adverse effect of the vanishing gradients can be ignored.

### Self-convolution based attention extractor module

In the traditional image field, self-convolution operation is an important method to calculate the similarity between two images. We aim to obtain the attention map about the intact rock region. To solve this problem, the self-convolution operation between the feature maps of the image $I$ and the feature maps of proposals $R_{i}$ has been introduced in this part. The feature map of proposals is the convolution kernel, while the feature maps of the image is the region to be convolved. It can help us get the attention map because of the two factors:

1. The value of the feature map to be convoluted. The response value will be larger when the value of the convolution kernel and the region to be convolved is larger.
2. The similarity between original image *I* and proposals region. The response value will be larger when the similarity is higher.

In general, the feature map value of conveyor belt and the clast region have small, and the similarity with the intact rock region is low. So we can get the more accurate attention map about the intact rock region.

**Figure 6:**
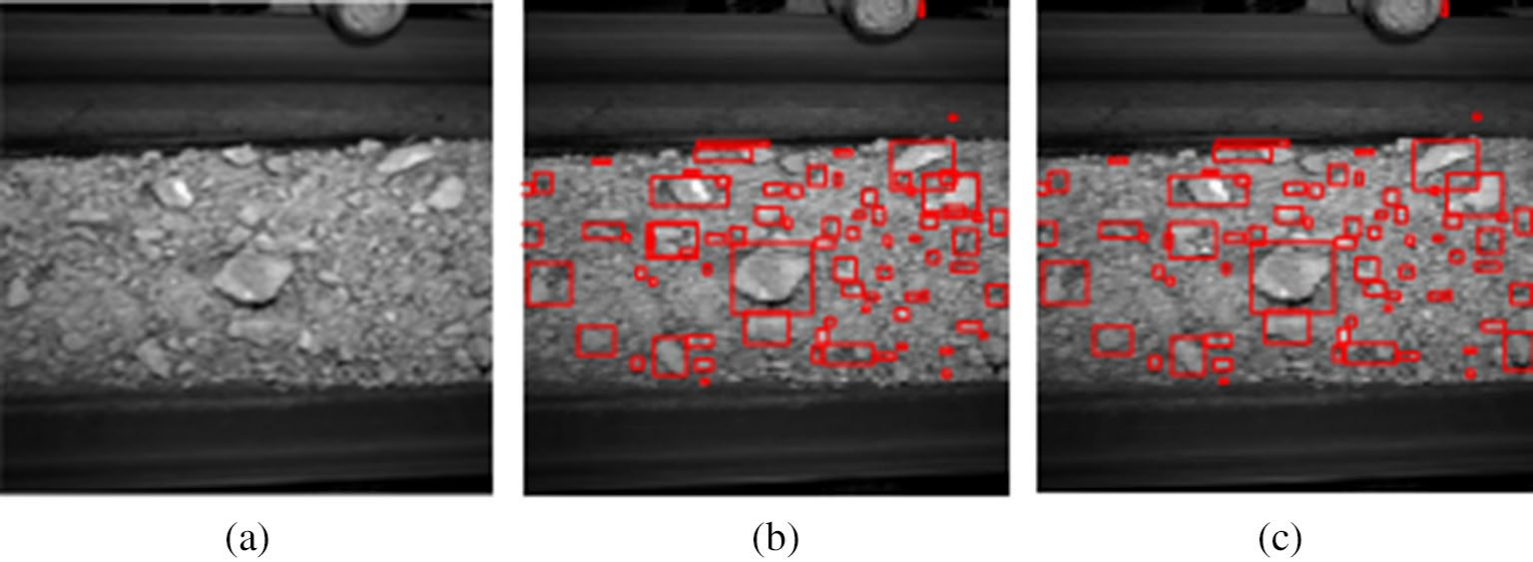
Selective search results. (**a**) Original image. (**b**) Selective search. (**c**) Repetition removal.

First of all, around 150 region proposals are extracted by the selective search^27^ on the original image (The selective search’s “fast mode” is utilized in this method on the original image (Fig. 6a) as shown in Fig. 6b. This method is implemented in two steps. First, the original image is initialized to get the small scale area by the segmentation method based on the graph theory. Then the large size area is merged considering the characteristics such as color, texture and computational complexity. The results show that the Mean Average Best Overlap is over 0.879.

The result of removing the repeat regions which the covered area between the region proposals are over 90% is shown in Fig. 6c.

**Figure 7:**
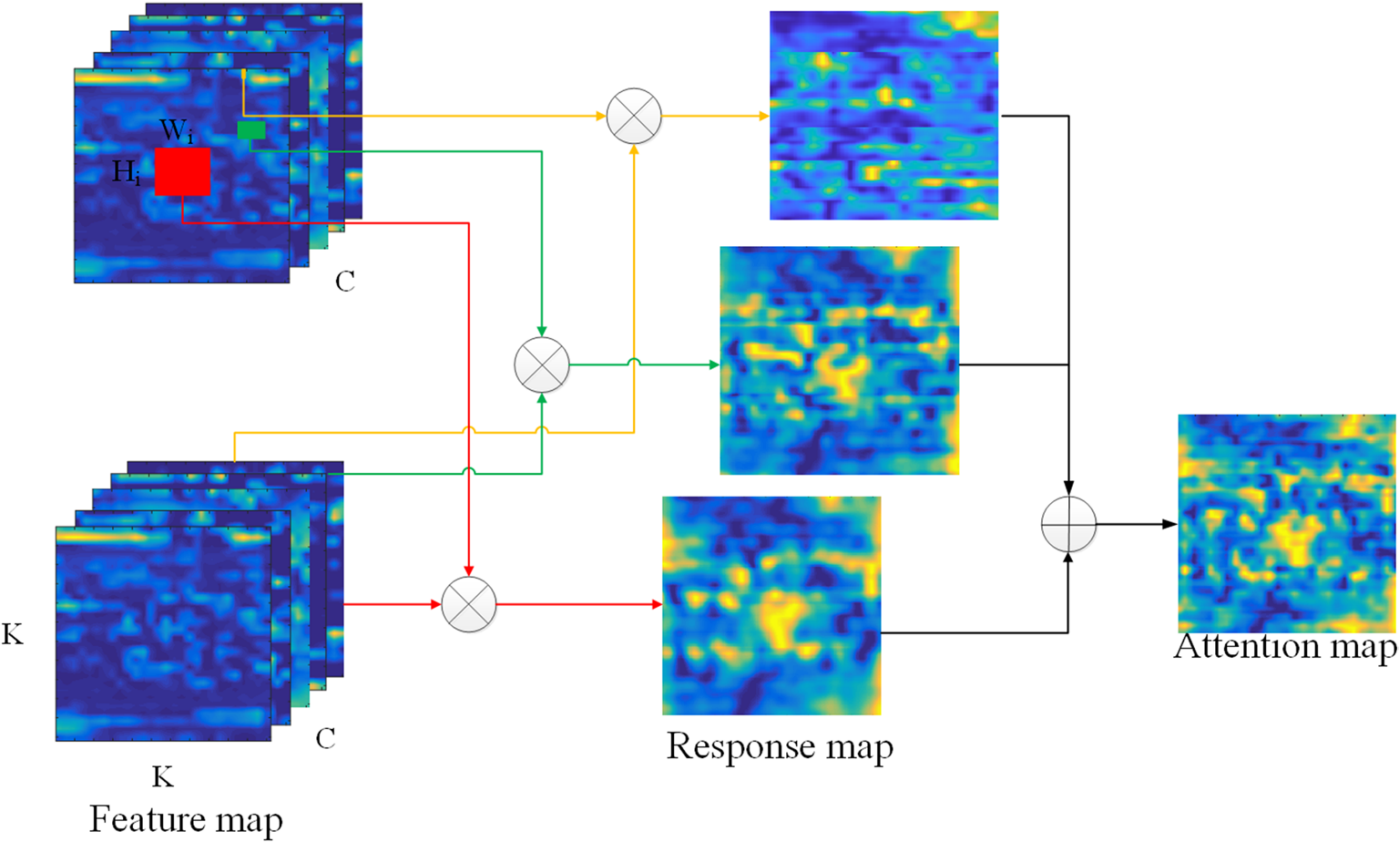
SAE module.

First, an original image *I* and its *N* proposals $R_{i}$ are inputted into the CNN baseline to get the corresponding feature map $F_{I}$ and $F_{R}$, with $F_{I} \subset {\mathbb{R}}^{C \times K \times K}$ and $F_{R} \subset {\mathbb{R}}^{{C \times H_{i} \times W_{i} }}$. Where the $H_{i}$(*K*),$W_{i}$(*K*), *C* indicate its rows, columns and channels of the feature map, respectively. Then, we choose the $x_{i} \in F_{R}$ as convolution kernel, while the $F_{I}$ is the region to be convolved. For any convolution kernel, the response map is calculated as follow:

2 $$S_{i} = x_{i} * F_{I} ,\quad i = 1,2, \ldots ,N,$$

where * is the self-convolution operation, which can be done with standard convolution. Figure 7 illustrates this proceed. For example, the yellow box region in Fig. 7 is the roller of the conveyor belt, so when the features of the yellow box region are used as the convolution kernel to deconvolve $F_{I}$, the response value of the roller region in the figure has a very high response value. While the red and green box regions are the intact rock region, it is easy to find that the focal regions of the response map are the intact rock regions. At the same time, because the red and green boxes correspond to rocks of different sizes, respectively, the response values of the response map obtained are also different for rock regions of different sizes.

Finally, there are *N* response maps after self-convolution operation. However, each response map can represent the results of only one local region. We should fuse all response map $x_{i}$ to obtain the final attention map *A*.

3 $$A = \frac{{e^{{\lambda \overline{S}}} }}{{\sum {e^{{\lambda \overline{S}}} } }},$$

where $\overline{S} = \frac{1}{N}\sum\nolimits_{i = 1}^{N} {S_{i} }$, $\lambda$ is the normalized hyperparameters of the softmax. The softmax normalization is to smooth the contribution of each region attention map.

### Self-convolution based attention pooling algorithm module

**Figure 8:**
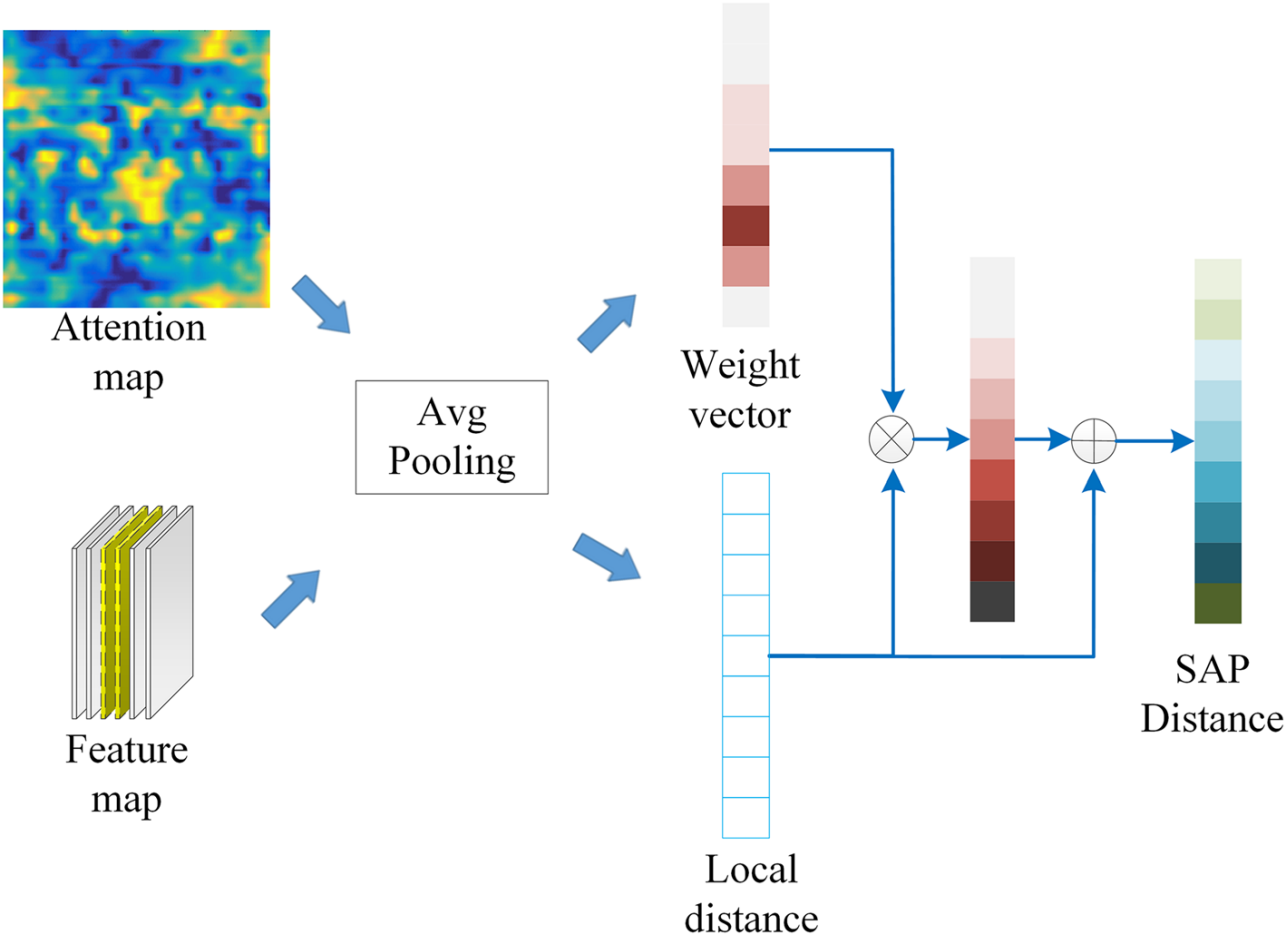
SAP module.

The attention map A is provided through the self-convolution operation in the previous section. In order to make the classification task more accurate, we proposed the self-convolution based attention pooling algorithm (SAP) as shown in Fig. 8.

First of all, the SAP utilizes the global average pooling to obtain the global average features of the image, and performs *L*_2_ normalization on the global average pooling. Then the distance between local features is calculated as follows:

4 $$d_{j} = \left\| {F_{I} - F_{C}^{j} } \right\|_{2}^{2} ,\quad j \in (1,2,3, \ldots ,9).$$

Then the weight vector $V = \left\{ {v_{1} ,v_{2} ,v_{3} , \ldots ,v_{9} } \right\}$ of the local feature is obtain by the global average pooling of attention map *A*. To the classification task, we can simply and directly take the local feature and weight vector progressive multiplication as the final classification distance vector. But this approach is problematic in dealing with the classification issue. The result gap in the final classification distance matrix becomes smaller. Therefore, we introduce the idea of residual to obtain SAP distance vector.

5 $$D_{j} = v_{j} d_{j} + d_{j} ,j \in (1,2,3, \ldots ,9)$$

Finally, the SAP distance vector is used to complete the classification task. The local feature of the intact rock regions is higher than other region. Because the response value of the attention map *A* is higher. Thus the effect of the intact rock area in the SAP distance is effectively enhanced.

In this paper, our proposed SFAN method includes two modules. The modules are integrated into the baseline network without breaking the baseline framework, making the modules easier to fuse with other deep learning networks.

## Experiment results

In this section, we first evaluate the performance of SAFN from aspects of interpretability, ablation, accuracy and cross-validation in the rock mass assessment dataset. Moreover, we conduct the dynamic test to analyze the assessment system based on SAFN model effective and real-time performance further.

### Dataset and implementation detail

Based on the assessment system, we select 12,600 images on nine types of rock to construct the rock mass assessment dataset. There are 900 train images and 500 value images in each category of the rock mass. The size of the input image is uniformly scaled to $448 \times 448$ pixels. We use standard mini-batch SGD, and adopt learning rate warm up as in Ref.^28^. The minimizing cross-entropy loss is select for classification task. In general, deep learning only works when there are a lot of data available. The change of camera angle and light intensity also require many samples. To enlarge the scale of the dataset, we adopt the data augmentation including random crop operation, random horizontal flip operation and etc.

### Evaluation metrics

We employed the accuracy (A), precision (P), recall (R), and F1-score (F_1_)^29^ for the online classification tasks, which are three widely used criteria to evaluate the superiority and applicability in pattern recognition.

6 $$A = \frac{{TP{ + }TN}}{{TP{ + }TN + FP + FN}},$$

7 $$P = \frac{TP}{{TP + FP}},$$

8 $$R = \frac{TP}{{TP + FN}},$$

9 $$F_{1} = \frac{2 \times P \times R}{{P + R}},$$

where true positive (TP) is the number of correct classified samples in the positive class, true negative(TN) is the number of false samples in the positive class, false positive (FP) is the number of false samples in the positive class, false negative (FN) is the number of false samples in the negatives class.

### Interpretability results of the attention map

In this section, we visualize the attention map and evaluate the Bbox localization error to evaluate the interpretability results of the self-convolution operation.

**Figure 9:**
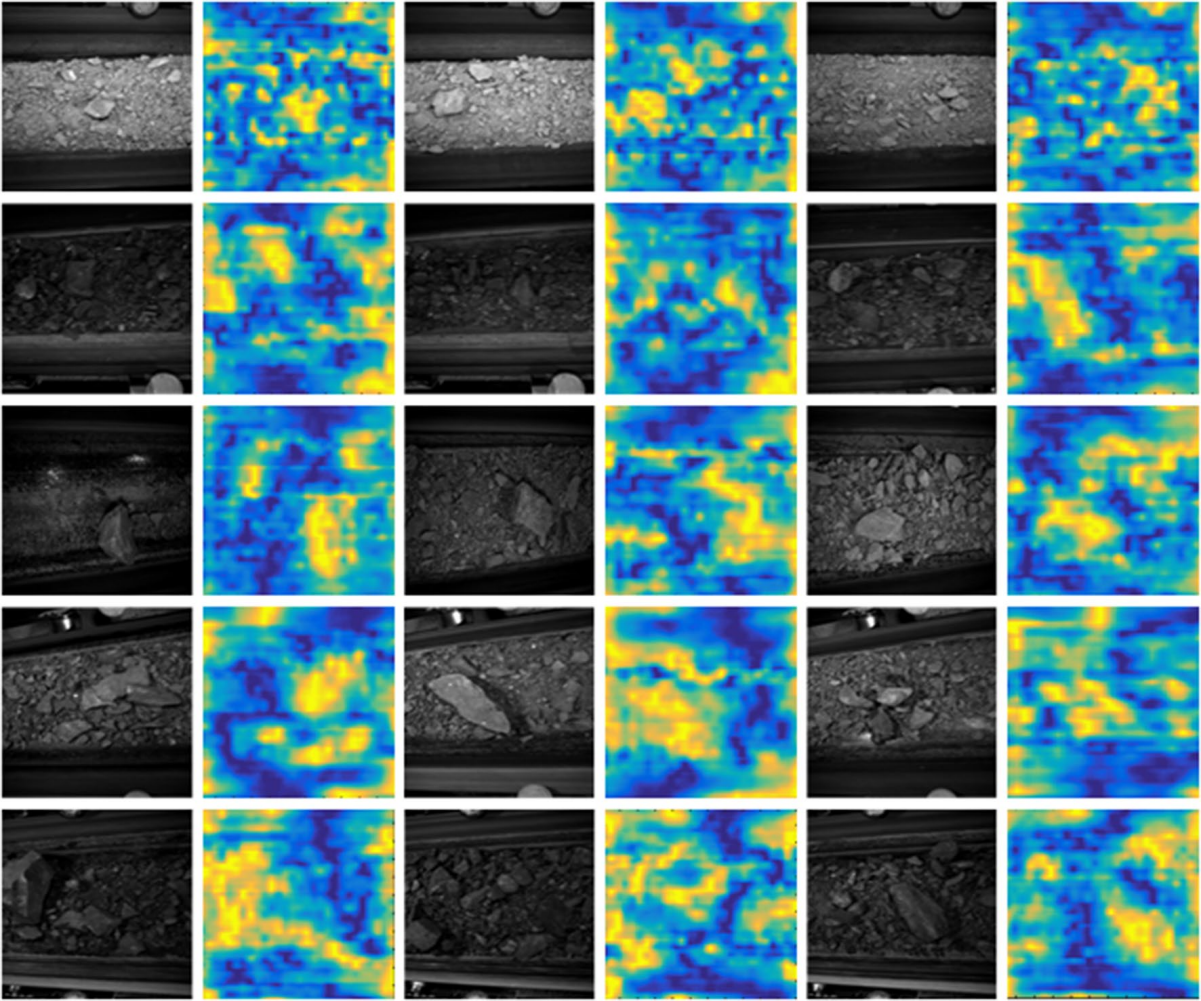
Example of attention map.

First, the visualized results of the attention map are shown in Fig. 9. We select the five types (GP, CS, MFS, AS, SM) rock as examples. Each line represents three same type rock mass original images and its attention map.

1. For the convolution kernel of the intact rock regions, its attention map can focus on the highly similar regions in the original image.
2. In the clast region or the background region, the response value of its attention map is suppressed. However, since the roller or other objects can be found by the selective search operation. There are some localization errors in the attention map.

These results indicate that the self-convolution operation can enable visual explanation that takes into account the rock information.

Then, we evaluate the bounding box (Bbox) localization error. The localization metric is suggested in Ref.^30^. In detail, if the Intersection-over-Union (IoU) of the rock mass is observed to be greater than 50% in the overlapped area between predicted Bbox and ground truth Bbox, the image bounding box can be considered as a correctly predicted label.

As shown in Table 1, we select the latest methods of GAP^31^ and CCAM^32^ to evaluate the Bbox localization errors. GAP utilizes the class activation map (CAM) to obtain target object regions. CCAM observes the activation maps from the highest to the lowest probability classes, and utilizes this attribute to suppress the background region, so as to achieve accurate object localization. Our model is achieving a 20.4% and 5.9% error reduction compared with GAP and CCAM, respectively. These qualitative results show that our model is able to select the intact rock regions for attribute recognition.

**Table 1:** Result of the Bbox localization errors.

Method	Localization error (%)
GAP	45.1
CCAM	30.6
SFAN	24.7

### Ablation experiment

In this paper, our proposed SFAN method includes two modules. One is the self-convolution based attention extractor module and the other is the self-convolution based attention pooling algorithm module. To investigate the influence of every module on rock mass assessment, we conduct the ablation test. We perform an ablation analysis of SAFN on the rock mass assessment dataset to evaluate how different components affect the detection performance. Table 2 shows the experimental results. First, the baselines are comparing in the 1st row (VGG network^33^) and the 4th row (ResNet network), the self-convolution based attention extractor module dramatically improves the accuracy as shown in the 2nd and 5th row. Also, there is no extra time introduced to the system. We find that our module makes the accuracy of ResNet-50 and VGG-19 improve at least 17.3% and 16.3%, respectively.

**Table 2:** Result of the ablation experiment.

Method	Network	Average of accuracy (%)	Time/image
VGG-19	VGG	68.6	56 ms
+ SAE	VGG	85.9	59 ms
SAFN	VGG	90.7	60 ms
ResNet-50	ResNet	71.9	51 ms
+ SAE	ResNet	88.2	53 ms
SAFN	ResNet	92.5	53 ms

Then, we verify the effectiveness of the SAP module. The accuracy of SAFN improves by 4.8% and 4.3%, as shown in the 3rd and 6th row. These results indicate that the SAP is slightly more accurate than SAE operation, which proves that the self-convolution based attention pooling algorithm can improve the model performance.

### Accuracy evaluation

In this section, we compare the accuracy results of ResNet-50 baseline with other state-of-the-art methods. The average accuracy and time cost per image on the rock mass assessment dataset are shown in Table 3. The most representative and advanced method are selected for the accuracy evaluation: WSCPM^34^, ABN^35^, ResNet-101^26^. The WSCPM detects the object part in a weakly supervised manner to build the complementary part. The classification task is complete by the local feature. The ABN introduces an attention-based branch structure to the classification network. The classification accuracy of WSCPM, ABN, ResNet-101 achieves 80.3%, 84.7% and 78.4%, respectively. Our model improves the accuracy by at least 7.8%. Compare with the state-of-art methods. We use the self-convolution operation instead of the network branch structure in WSCPM and ABN. Our method has better advantages in terms of time.

**Table 3:** Result of the accuracy evaluation.

Method	Average of accuracy (%)	Time/image
WSCPM	80.3	110 ms
ABN	84.7	167 ms
ResNet-50	71.9	51 ms
ResNet-101	78.4	59 ms
SAFN	92.5	53 ms

### Cross-validation of rock mass assessment dataset

To further explore the SAFN model, the confusion matrix is used to evaluate the classification results. The confusion matrix is a widely used index for recognition evaluation. Each column represents the predicted category, and the total number of each column represents the number of data classified into categories. Each row represents the actual category to which the data belongs, and the total number of data in each row represents the number of data instances of that category. The confusion matrix is calculated by summing up the total number of observation accuracy values of the false and correct categories in the statistical recognition model. Table 4 shows the confusion matrix obtained in the rock mass assessment dataset using the SFAN model. GP is with an accuracy of 95.77%, which is the easiest to be accurately identified in the rock mass assessment, followed by SM, CB, AS, BL, CS, TB, ASL and MFS with accuracies of 94.72%, 93.18%, 92.42%, 92.12%, 91.8% 91.29%, 91.05%, 90.05%, respectively. Furthermore, it can be inferred that among the 9 categories, TB is easy to be misclassified as CB, since its size and distribution of rock mass is located closer to CB.

**Table 4:** Confusion matrix of classification results by SAFN model.

Rock structure type	GP	CS	MFS	AS	SM	TB	CB	ASL	BL
Granite porphyry (GP)	95.77%	0.84%	1.27%	0.42%	0.12%	0.07%	0.10%	0.47%	0.94%
Conglomeratic sandstone (CS)	0.76%	91.80%	3.58%	1.04%	1.28%	0.26%	0.18%	1.08%	0.02%
Medium fine sandstone (MFS)	0.82%	3.08%	90.05%	3.17%	1.19%	0.05%	0.11%	1.21%	0.32%
Argillaceous siltstone (AS)	0.47%	1.95%	2.56%	92.42%	1.24%	0.45%	0.66%	0.14%	0.11%
Silty mudstone (SM)	0	0.95%	1.04%	3.06%	94.72%	0.02%	0	0	0.21%
Tectonic breccia (TB)	1.34%	0.42%	0.26%	0.81%	0.31%	91.29%	3.41%	0	2.16%
Cryptoexplosive breccia (CB)	0.73%	0.04%	0.14%	0.05%	0.03%	4.82%	93.18%	0	1.01%
Argillaceous silty limestone (ASL)	0.08%	1.05%	1.84%	0.32%	1.21%	0.13%	0.08%	91.05%	4.24%
Bioclastic limestone (BL)	1.37%	0.24%	0.32%	0.13%	0.59%	1.04%	1.56%	2.63%	92.12%

**Figure 10:**
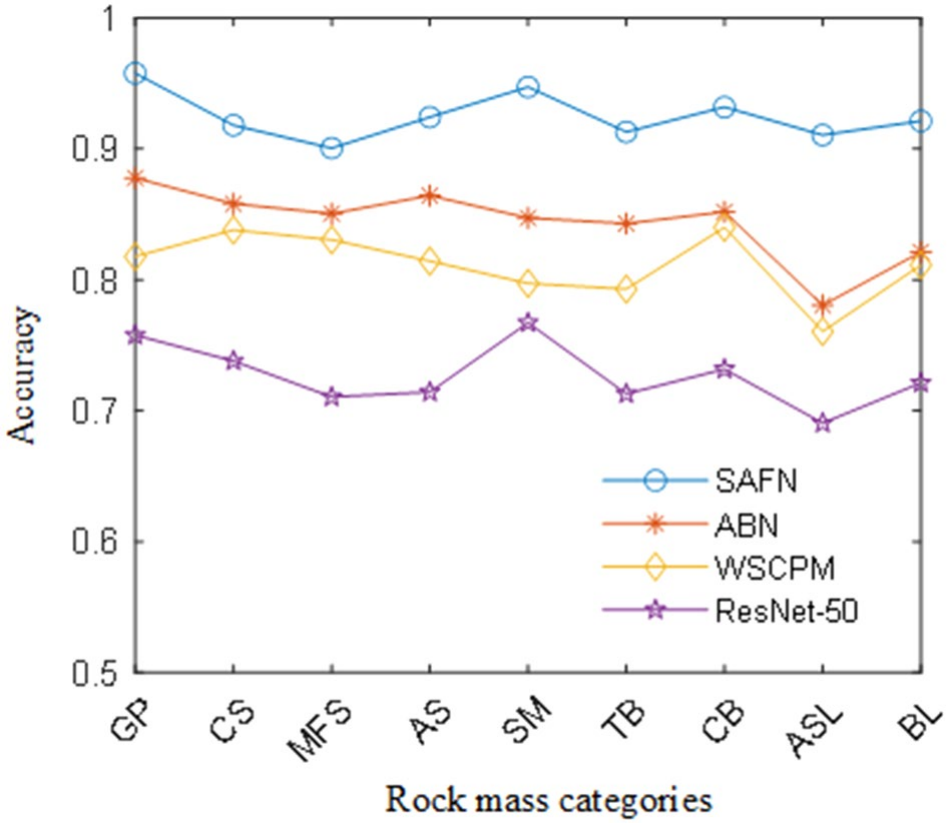
Geological distribution of the sampling site.

Additionally, the comparison experiment between the four methods for different categories rock mass is shown in Fig. 10. It can be seen that all the evaluation metrics of CNN models present a similar trend. Overall, the values of the metrics from the highest to the lowest appear in this trend: SM, CB, BL, CS, TB, MFS, AS, and ASL. Since the texture features and distinct appearance of the GP images, it makes the all the methods corresponding more prominent in GS identification. The ASL and AS images in the dataset have many similar features. Therefore, the classification of ASL and AS images suggest relatively poor performance. However, our method focuses on the intact rock details and has better performance at distinguishing AS from ASL. This also proves that our method has great advantages in the classification of similar rocks.

### Dynamic test for the assessment system

**Figure 11:**
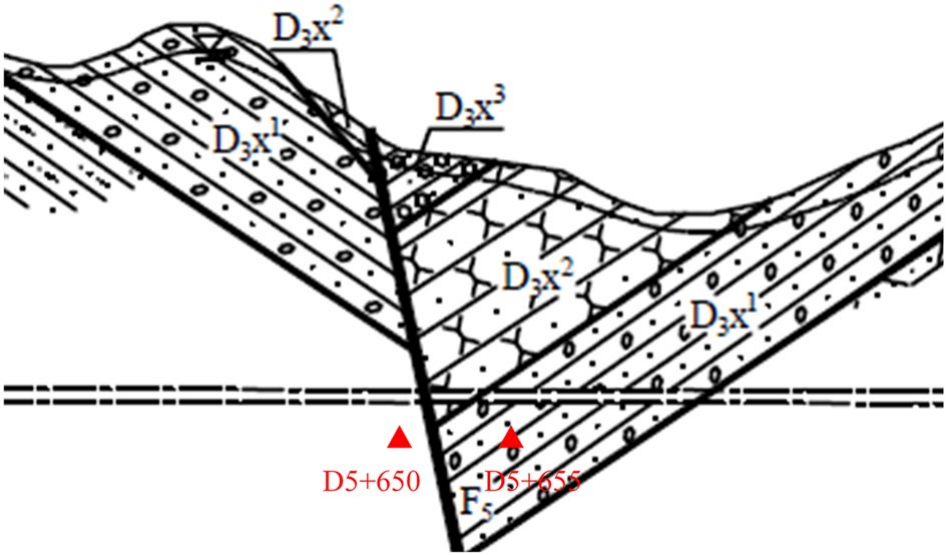
Geological distribution of the sampling site.

**Figure 12:**
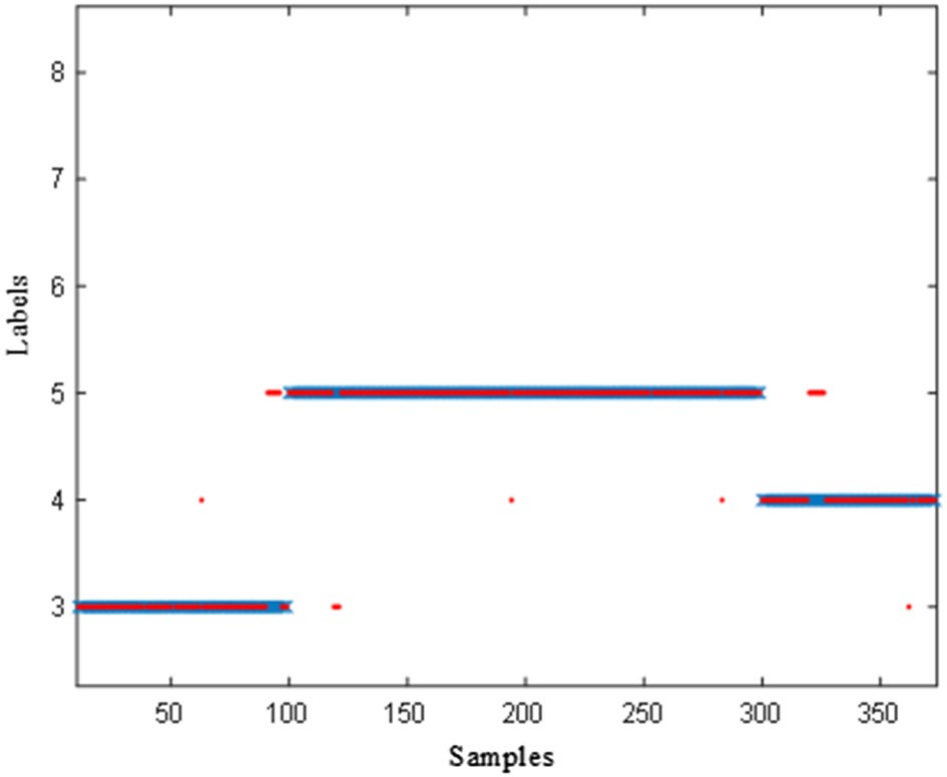
Classification results for the dynamic test.

To verify the performance of the SAFN model in application, the classification dynamic test with the assessment system based on the SAFN model are conducted in the section of D5 + 650 to D5 + 655 in Hangzhou Second Source Water Conveyance Channel Project (Shanling section, Jiangnan Route), Hangzhou, China as shown in Fig. 11. There are 400 online samples in the three types of geological conditions during this section: MFS, SM and AS. The real-time classification results for the dynamic test are shown in Fig. 12.

The classification results are shown in Table 5. The precision of the MFS, SM and AS reached to 94.8%, 94.7% and 96.7%, respectively. Overall, the dynamic test indicates that our system is efficient for automated rock mass classification.

**Table 5:** Result of the dynamic test.

	Precision	Recall	F_1_
Medium fine sandstone (MFS)	94.8%	91%	92.9%
Silty mudstone (SM)	94.7%	97%	95.8%
Argillaceous siltstone (AS)	96.7%	88%	92.1%

### Analysis of real-time performance

The real-time performance is crucial for the assessment system based on SAFN model. We evaluate the inference speed of the system. This experiment was implemented on one NVIDIA GeForce RTX 3090 GPU. The data upload time is 300 ms. The inference speed of the SAFN model under image resolution of $1024 \times 1024$ achieves 132 ms, under image resolution of $448 \times 448$ achieves 53 ms and under image resolution of $224 \times 224$ achieves 17 ms. In general, the mean run time of a rock image is less than 1 s.

## Conclusion and future work

In this research, we construct a visual assessment system, install it on the TBM. Then, we collect a larger in-situ rock mass image dataset from the construction site of the TBM. It includes around 12,600 rock mass images covering nine different rock types. This dataset is the first attempt to classify the excavated rock during TBM tunnel excavation. To assess the rock mass, we have presented a self-convolution based attention fusion network for rock mass assessment. The core of our method is the discovery and fusion of the object attention map within a deep neural network. We focus on the attention map not only for the region of interest (ROI) extraction, but also for improving the performance of CNN by fusing it that focuses on the intact rock regions in an image. First, SAFN detects the intact rock regions in the image by the SAE module. Then, the SAP module is proposed in the classifier, which is trainable for image recognition in an end-to-end manner. We integrate a region-based part attention map into the deep network through the SAP module.

To evaluate the SAFN model, we conduct extensive experiments to indicate the accuracy, interpretability and efficiency of the SFAN model in the rock mass assessment dataset. Moreover, the dynamic test shows that our assessment system based on the SAFN model is accurate and efficient for automated classification of rock mass during TBM tunnel excavation. However, establishing an automatic driving system for TBM based on the rock mass assessment proposed in this paper remains a challenging task.

## References

[1] Cho JW, Jeon S, Yu SH, Chang SH (2010). Optimum spacing of tbm disc cutters: A numerical simulation using the threedimensional dynamic fracturing method. Tunn. Undergr. Space Technol..

[2] Gong Q, Yin L, Ma H, Zhao J (2016). TBM tunnelling under adverse geological conditions: An overview. Tunn. Undergr. Space Technol..

[3] Zhang W, Han L, Gu X, Wang L, Chen F, Liu H (2020). Tunneling and deep excavations in spatially variable soil and rock masses: A short review. Undergr. Space.

[4] Alippi C, Boracchi G, Roveri M (2016). A reprogrammable and intelligent monitoring system for rock-collapse forecasting. IEEE Syst. J..

[5] Xue Z, Chen L, Liu Z, Lin F, Mao W (2021). Rock segmentation visual system for assisting driving in TBM construction. Mach. Vis. Appl..

[6] Hassanpour J, Rostami J, Khamehchiyan M (2010). TBM Performance analysis in pyroclastic rocks: A case history of Karaj water conveyance tunnel. Rock Mech. Rock Eng..

[7] Hassanpour J, Rostami J, Zhao J (2011). A new hard rock TBM performance prediction model for project planning. Tunn. Undergr. Space Technol..

[8] Adoko AC, Yagiz S (2019). Fuzzy inference system-based for TBM field penetration index estimation in rock mass. Geotech. Geol. Eng..

[9] Liu B, Wang R, Zhao G, Guo X, Wang Y, Li J, Wang S (2020). Prediction of rock mass parameters in the TBM tunnel based on BP neural network integrated simulated annealing algorithm. Tunn. Undergr. Space Technol..

[10] Song F, Wang H, Jiang M (2018). Analytically-based simplified formulas for circular tunnels with two liners in viscoelastic rock under ani-sotropic initial stresses. Constr. Build. Mater..

[11] Su K, Zhang Y, Chang Z, Wu H, Wang T, Zhou W (2019). Transverse extent of numerical model for deep buried tunnel excavation. Tunn. Undergr. Space Technol..

[12] Xie H, Jiang W, Luo H, Yu H (2021). Model compression via pruning and knowledge distillation for person reidentification. J. Ambient Intell. Hum. Comput..

[13] Li L, Liu G, Zhang L, Li Q (2021). FS-LSTM-based sensor fault and structural damage isolation in SHM. IEEE Sens. J..

[14] Chen J, Yang T, Zhang D, Huang H, Tian Y (2021). Deep learning based classification of rock structure of tunnel face. Geosci. Front..

[15] Gu J, Wang Z, Kuen J, Ma L, Shahroudy A, Shuai B, Liu T, Wang X, Wang G, Cai J, Chen T (2018). Recent advances in convolutional neural networks. Pattern Recogn..

[16] Gong QM, Zhao J, Jiao YY (2005). Numerical modeling of the effects of joint orientation on rock fragmentation by tbm cutters. Tunn. Undergr. Space Technol..

[17] Chen L, Li Y, Zhong X, Zheng Q, Liu H (2019). An automated system for position monitoring and correction of chord-based rail corrugation measuring points. IEEE Trans. Instrum. Meas..

[18] Song, L., Gong, D., Li, Z., Liu, C., Liu, W. Occlusion robust face recognition based on mask learning with pairwise differential siamese network. In *ICCV*, 773–782 (2019).

[19] Vinayahalingam S, Kempers S, Limon L, Deibel D, Maal T, Hanisch M, Bergé S, Xi T (2021). Classification of caries in third molars on panoramic radiographs using deep learning. Sci. Rep..

[20] Dwivedi R, Dey S, Chakraborty C, Tiwari S (2021). Grape disease detection network based on multi-task learning and attention features. IEEE Sens. J..

[21] Song, S., Zhang, W., Liu, J., Mei, T. Unsupervised person image generation with semantic parsing transformation. In *CVPR*, 2352–2361 (2019). 10.1109/TPAMI.2020.2992105 32365019

[22] Zhu Q, Zhong Y, Zhao B, Xia G, Zhang L (2016). Bag-of-visual-words scene classifier with local and global features for high spatial resolution remote sensing imagery. IEEE Geosci. Remote Sens. Lett..

[23] Li W, Liang Y, Zhang X, Liu C, He L, Miao L, Sun W (2021). A deep learning approach to automatic gingivitis screening based on classification and localization in RGB photos. Sci. Rep..

[24] Selvaraju RR, Cogswell M, Das A, Vedantam R, Parikh D, Batra D (2020). Grad-CAM: Visual explanations from deep networks via gradient-based localization. Int. J. Comput. Vis..

[25] Fan X, Jiang W, Luo H, Fei M (2019). SphereReID: Deep hypersphere manifold embedding for person re-identification. J. Vis. Commun. Image Represent..

[26] He, K., Zhang, X., Ren, S., Sun, J. Deep residual learning for image recognition. In *CVPR*, 770–778 (2016)

[27] Uijlings JRR, van de Sande KEA, Gevers T, Smeulders AWM (2013). Selective search for object recognition. Int. J. Comput. Vis..

[28] Luo H, Jiang W, Gu Y, Liu F, Liao X, Lai S, Gu J (2020). A strong baseline and batch normalization neck for deep person re-identification. IEEE Trans. Multimed..

[29] Liu J, Teng Y, Ni X, Liu H (2021). A Fastener inspection method based on defective sample generation and deep convolutional neural network. IEEE Sens. J..

[30] Russakovsky O, Deng J, Su H, Krause J, Satheesh S, Ma S, Huang Z, Karpathy A, Khosla A, Bernstein M, Berg AC, Fei-Fei L (2015). Imagenet large scale visual recognition challenge. Int. J. Comput. Vis..

[31] Zhou, B., Khosla, A., Lapedriza, À., Oliva, A., Torralba, A. Learning deep features for discriminative localization. In *CVPR*, 2921–2929 (2016).

[32] Yang, S., Kim, Y., Kim, Y., Kim, C. Combinational class activation maps for weakly supervised object localization. In *WACV* (2020)

[33] Simonyan, K. & Zisserman, A. Very deep convolutional networks for large-scale image recognition. https;//arXiv.org/abs/1409.1556 (2014).

[34] Ge, W., Lin, X., Yu, Y. Weakly supervised complementary parts models for fine-grained image classification from the bottom up. In *CVPR*, 3029–3038 (2019).

[35] Quintino Ferreira, B., Costeira, J. P., Sousa, R. G., Gui, L., Gomes, J. P. Pose guided attention for multi-label fashion image classification. In *ICCV Workshop*, 3125–3128 (2019).

